# Acute Hemorrhagic Myositis in Inflammatory Myopathy and Review of the Literature

**DOI:** 10.1155/2014/639756

**Published:** 2014-10-14

**Authors:** Howard Van Gelder, Kim M. Wu, Nayiri Gharibian, Dharmi B. Patel, Philip J. Clements, Emil R. Heinze, Robert I. Morris, Andrew L. Wong

**Affiliations:** ^1^UCLA-Olive View Rheumatology Program, Division of Rheumatology, Olive View-UCLA Medical Center, 14445 Olive View Drive, 2B182, Sylmar, CA 91342, USA; ^2^UCLA-Olive View Internal Medicine Program, Department of Medicine, Olive View-UCLA Medical Center, 14445 Olive View Drive, 2B182, Sylmar, CA 91342, USA

## Abstract

We describe two patients with dermatomyositis that presented with interstitial lung disease, positive V and Shawl sign who developed acute spontaneous abdominal/retroperitoneal bleed. Both patients expired despite aggressive treatment and resuscitation. Hemorrhagic myositis in these two patients with inflammatory myopathy is a very rare complication. The association of anti-Ro52 with this potentially very serious complication remains unclear. This potential relationship should be further evaluated in future studies.

## 1. Introduction

Dermatomyositis is an autoimmune idiopathic inflammatory myopathy (IIM) with characteristic skin manifestations, classically heliotrope rash, V, shawl, and Holster sign, and Gottron's papules [1]. Up to 30 percent of patients with dermatomyositis (DM) or polymyositis (PM) have a constellation of clinical findings termed the “antisynthetase syndrome” [2–4]. Findings include constitutional symptoms such as fever, interstitial lung disease (ILD), myositis, Raynaud's phenomenon, mechanic's hands, and arthritis [5]. Dermatomyositis can be associated with systemic manifestations including ILD, pneumomediastinum, and cardiomyopathy [6]. We present two cases of inflammatory myopathy (Dermatomyositis) complicated by acute spontaneous hemorrhage of the iliopsoas, psoas, and pectineus muscles. To our knowledge, there are only a handful of cases of myositis complicated by hemorrhage.

## 2. Case 1

A 50-year-old African-American male with a history of borderline diabetes presented with three weeks of myalgia, a fifteen-pound weight loss, arthralgia, odynophagia, fatigue, dyspnea on exertion, and progressive nonproductive cough in early 2012. These symptoms coincided with a new erythematous rash on the back of his scalp, neck, and inner left ear and over the knuckles of his hands. A short trial of 4 mg of oral methylprednisolone was given at a previous urgent care visit with some improvement in the rash. Review of systems confirmed intermittent subjective fever, hoarseness, and tea color urine for 4 days. Medications included over-the-counter smooth move for constipation, multivitamins, and fish oil.

Initial exam showed normal vital signs. His voice was hoarse. His lung exam revealed mild crackles bilaterally. Muscle strength testing of the neck flexors and proximal upper and lower extremities was 4/5 with bilateral distal upper and lower extremities scoring 5/5. Skin exam showed malar erythema, erythematous patches over the scalp and behind the ears, minimal heliotrope rash, mild erythema over his chest (V sign), and diffuse erythematous hypopigmented macules over his proximal interphalangeal joints (PIPs) and metacarpal phalangeal joints (MCPs) bilaterally. He had “mechanic's hands” and periungal erythema. There was a small hard papule along the medial aspect of the right 3rd PIP, which was suspected of being calcinosis.

**Figure 1:**
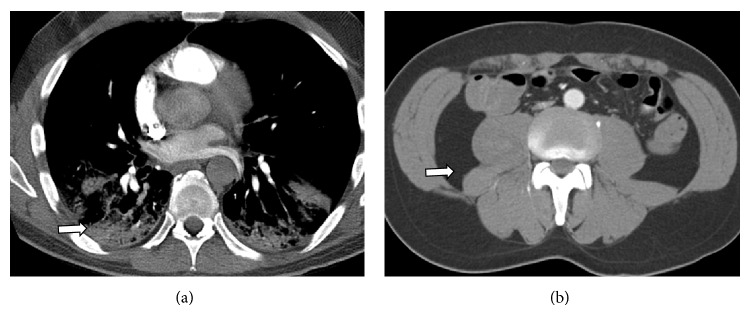
(a) CT chest on admission. Axial contrast enhanced images through the chest in the pulmonary phase demonstrate a normal main pulmonary artery trunk and normal segmental pulmonary arterial branches extending to the superior segment of the right upper lobe. Lung windows reveal lower lobe consolidation (see arrow). (b) Normal appearance of the retroperitoneum at that time (see arrow).

Initial laboratory results revealed elevated creatine phosphokinase (CPK) of 1123 units/L (reference: 26–174 units/L) with mild elevation of transaminases but unremarkable complete blood count (CBC), chemistry panel, and coagulation studies. Chest X-ray showed patchy consolidations of the right upper lung and posterior lung bases suggestive of possible pneumonia. CT chest showed right upper lobe ground-glass opacity and bilateral lower lobe consolidations with mildly enlarged mediastinal lymph nodes (Figure 1). He was started on ceftriaxone and azithromycin for empiric coverage of community acquired pneumonia. He was admitted for treatment and workup of a possible inflammatory myopathy.

**Figure 2:**
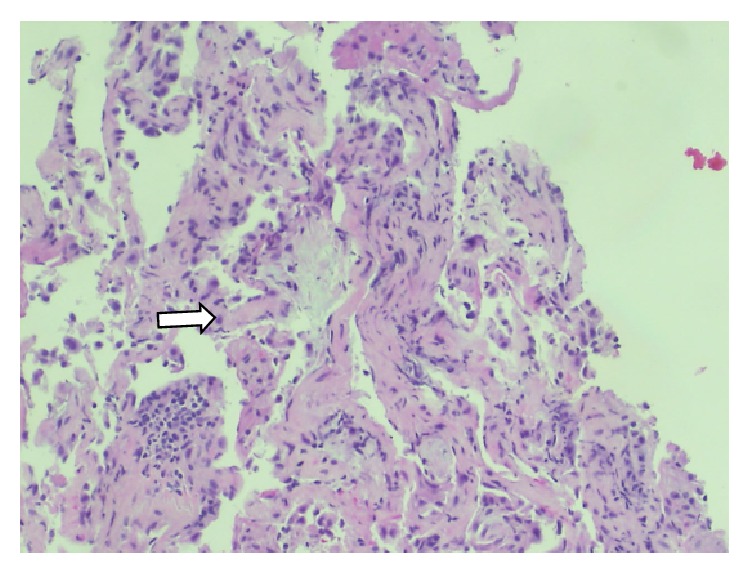
Lung biopsy (mild chronic interstitial pneumonitis with focal fibroblastic foci, consistent with organizing pneumonia; stains for fungi and AFB were negative) (see arrow).

During the initial hospitalization, the patient's CPK improved with bed rest and normal saline infusion. CT abdomen and pelvis ordered to evaluate persistent constipation was unremarkable except for a small hepatic cyst. EMG/NCS showed no electrodiagnostic evidence of a polyneuropathy or myopathy. MRI-STIR of the thighs showed no convincing evidence of an inflammatory myopathy other than minimal muscle edema of the iliopsoas muscles. Given no clear focus of inflammatory muscle seen on MRI or EMG, a muscle biopsy was deferred. For continued hoarseness of voice, laryngoscopy was performed and showed postcommissure edema and erythema involving the laryngeal epiglottis and false cords. For evaluation of continuing dyspnea with gradual worsening hypoxia, a bronchoscopy was performed that visualized signs of mild inflammation in the lungs. Bronchoalveolar lavage returned numerous WBCs with normal respiratory flora present. Transbronchial biopsy of the right lower lung was consistent with organizing pneumonia and was negative for fungi and acid fast bacilli (Figure 2). A transthoracic echocardiogram was read as within normal limits and a CT angiogram was negative for pulmonary embolism. Immunofluorescence for antinuclear antibodies (ANA), serum for anti-RNP/Sm, anti-phospholipid antibodies, anti-Mi-2, anti-SRP, anti-Jo-1, anti-neutrophil cytoplasmic antibodies (ANCA), HIV, hepatitis panel, aldolase, and sputum for AFBx3 all returned normal.

**Figure 3:**
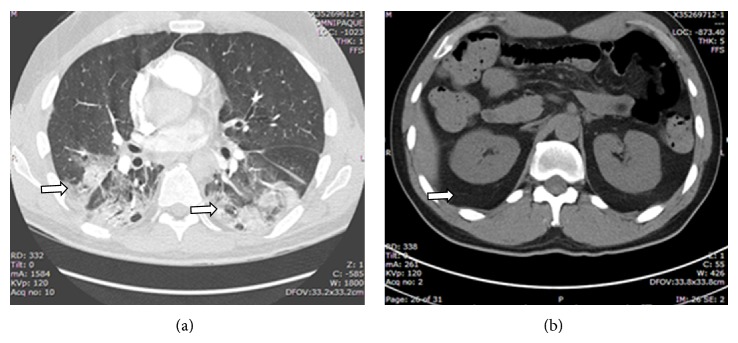
(a) CT chest/abdomen/pelvis. No evidence for pulmonary embolism. Patchy peribronchiolar consolidations are worsened in the right upper lobe, right lower lobe, and left lower lobe (see arrow). (b) No abdominal masses/hemorrhage is seen (see arrow).

**Figure 4:**
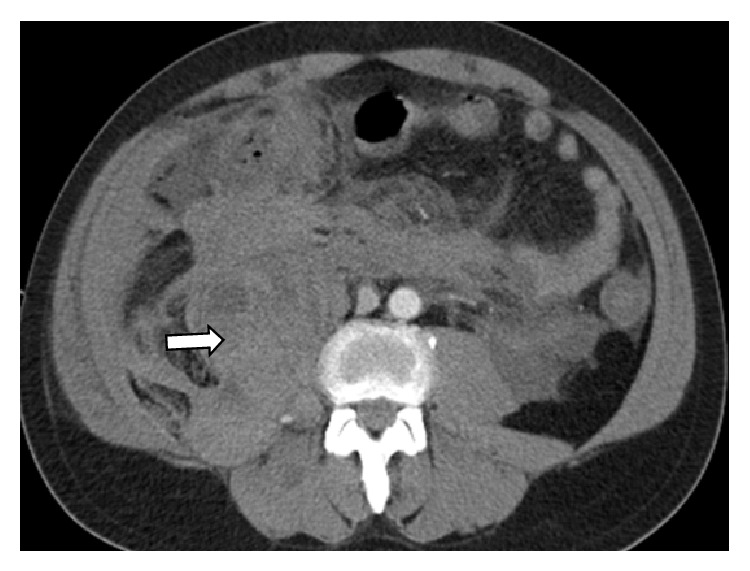
Stat CT abdomen/pelvis. Interval development of a large retroperitoneal hemorrhage centered around the right psoas muscle, with extension to the right spinous erector and iliacus musculature (see arrow).

In the interim, the patient developed persistent fever thought likely to be secondary to an underlying inflammatory process. Solumedrol 60 mg IV q 12 hours was started after one week into his hospitalization with near immediate resolution of fevers and improvement of the rash. Given the likely diagnosis of dermatomyositis, two doses of intravenous immunoglobulin (IVIG) were given to treat his persistent dysphagia and unresolving organizing pneumonia. Patient reported improved strength after receiving IVIG and steroid therapy, but he remained hypoxic. Repeat CT scans of chest/abdomen/pelvis were performed and showed no progression of disease on day 15 (Figure 3). On day 17 of hospitalization, the patient began complaining of right hip pain for which an MRI was ordered to rule out avascular necrosis. Later that evening, the patient was found to be uncomfortable in bed and only partially responsive. He became hypotensive, tachycardic, and tachypneic. CBC revealed hemoglobin of 4.1 gm/dL and platelet count of 50,000/*μ*L. The patient was intubated, started on vasopressors, and transferred to the intensive care unit (ICU). He was aggressively resuscitated with IV fluid and blood products. He was also noted to have increasing abdominal girth. Stat CT abdomen/pelvis showed large retroperitoneal bleeding involving the right iliacus and psoas muscles, extending into the retroperitoneum and along the right paracolic gutter (Figure 4). Interventional radiology could not locate a focal source of bleeding and suggested aggressive medical resuscitation. Solumedrol was also increased to 250 mg IV Q6 for possible dermatomyositis associated vasculitis. The patient received a total of 20 units of pack red blood cells, fresh frozen plasma, cryoprecipitate, and 3 units of platelets but ultimately progressed into multiorgan failure. On day 19, the patient was found to be in asystole and expired that day.

**Figure 5:**
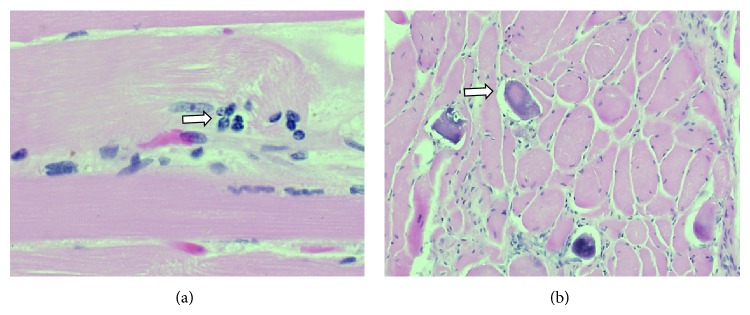
(a) Autopsy of the psoas muscle. Skeletal muscles: nonuniform degree of interior scattered segments of necrotic myofibers in the psoas fascicles without perifascicular myofiber atrophy or necrosis. Extensive morular aggregation of myofiber nuclei in the damaged myofibers (see arrow). Minimal perimysial and endomysial fibrosis, suggesting an acute process. Finding may suggest possible sepsis with intravascular coagulation and capillary disruption. (b) Calcium deposition found in myofiber (see arrow).

The family subsequently agreed to a partial autopsy limited to the chest and abdomen. The autopsy results showed a massive retroperitoneal hematoma, segmental myofiber necrosis in the psoas muscle without lymphocytic infiltrate, and perifascicular atrophy or necrosis (Figure 5). The patient's serum was sent to a reference lab where anti-Ro52 returned positive.

## 3. Case 2

A 63-year-old Filipino female with a history of diabetes, hypertension, and hyperlipidemia presented to our hospital for new throat pain, fevers, and oral sores for four weeks in early 2013. She had difficulty swallowing to the point where she could no longer eat solid foods and had lost 10–15 lbs. She developed new painful mouth sores involving her lips along with low grade subjective fevers and joint swelling of the fingers during the same time period. She reported a new rash around her eyes and chest that was not itchy or painful. She complained of worsening fatigue over the past week, required assistance to eat and go to the bathroom, and was bedbound on presentation. ROS confirmed a dry cough for four weeks not improved with oral azithromycin prescribed by her PMD. She had been seen in the ED one month ago for the joint pains where a hand XR reportedly showed osteoarthritis of her hands and a chest X-ray noted prominent interstitial lung markings. Medications included amlodipine, metformin, quinapril, ezetimibe, glipizide, aspirin, calcium, and fish oil.

**Figure 6:**
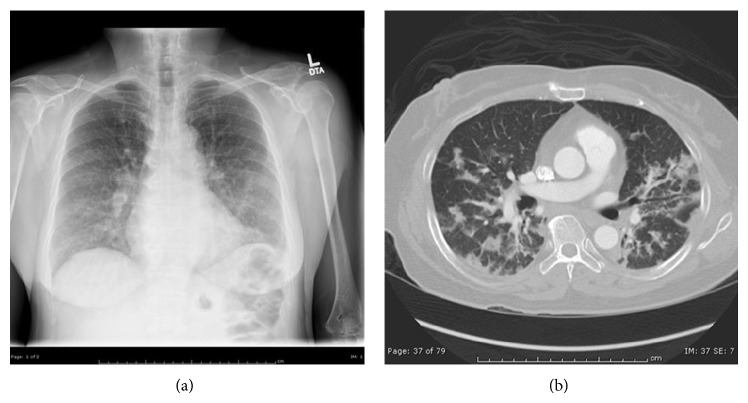
Lung imaging on admission. (a) Chest radiograph showing prominent interstitial lung markings. (b) CT pulmonary angiogram showing multifocal opacities and consolidation.

Upon presentation she was afebrile with a blood pressure of 140/80 mmHg, pulse of 107 beats/minute, respiratory rate of 26 breaths/minute, and oxygen saturation of 86% on room air that improved to 95% with 2 L nasal cannula oxygen. She was found to be severely hyponatremic with poor PO intake and have a new bilateral infiltrate on CT angiogram (Figure 6) of the chest, and thus she was admitted for further care. Initial exam was significant for hoarse voice, oropharyngeal ulcerations/erythema, heliotrope rash around the eyes, positive shawl and V sign, and bilateral crackles at the lung bases. Her hand examination showed distal cracking of her skin (mechanic's hands) along with purpuric lesions. Strength testing showed initially 5/5 in all muscle groups but decreased in the lower extremities to 3/5 early in the course of her hospital stay.

**Figure 7:**
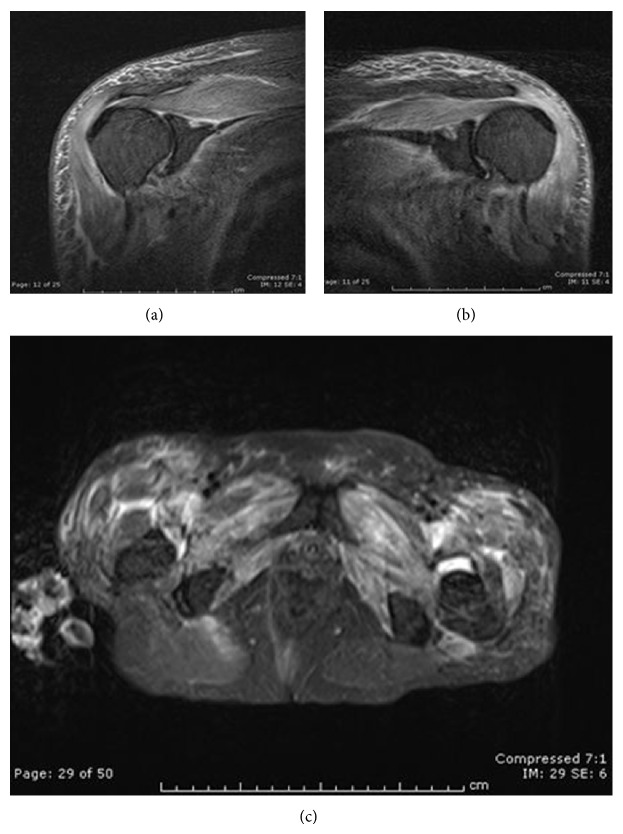
MRI with short tau inversion recovery (STIR) image showing diffuse symmetrical proximal muscle edema. (a) Right shoulder, (b) left shoulder, and (c) pelvis.

Initial labs were significant for CPK level of 2246 IU/I, aldolase of 14.2 IU/L, LDH 498 IU/L, AST 310 IU/L, ALT 77 IU/L, alkaline phosphatase 309 IU/L, T. bilirubin 0.7 mg/dL, and CRP 41.5 mg/mL. All testing for bacterial/fungal pneumonia, urine histoplasmosis antigen, viral hepatitis antibody/antigens, aspergillosis antigen, legionella antigen, influenza A/B antibody, syphilis (RPR) antibody, coccidioidomycosis antibody, and cryptococcus antibody was negative. ANA, anti-RNP/Sm, anti-dsDNA, anti-Scl70, anti-centromere B, anti-smooth muscle, anti-histone, anti-CCP, ANCA, anti-Mi-2, anti-Jo-1, and anti-SSB antibodies were all negative. Anti-SSA was mildly positive at >1.2 (normal < 1.0 IU). Magnetic resonance imaging (MRI) with short tau inversion recovery (STIR) imaging of the pelvis and shoulders showed diffuse edema in symmetric distribution throughout the shoulder girdles and pelvis bilaterally (Figure 7). Electromyogram with nerve conduction study revealed sharp waves and irritable myopathy consistent with inflammatory myopathy.

**Figure 8:**
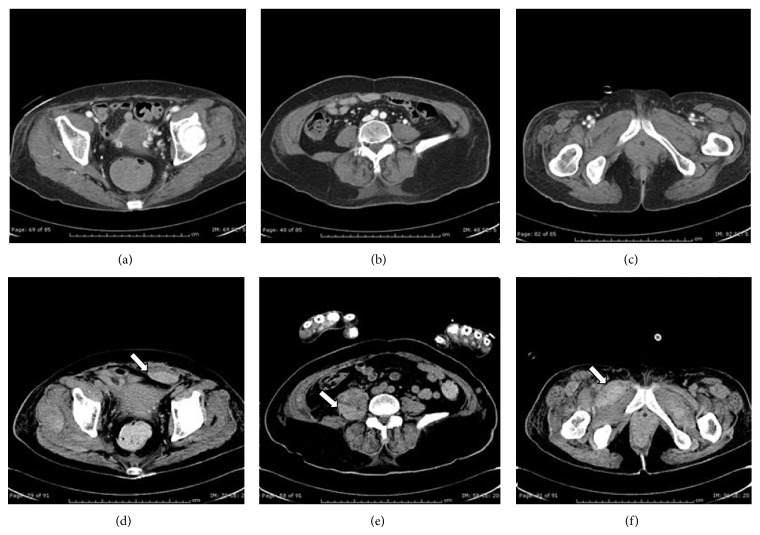
CT scan of the abdomen and pelvis before and after bleeding. (a–c) CT scan with contrast shows (a) normal rectus, (b) normal iliopsoas, and (c) normal pectineus muscles. (d–f) CT scan without contrast shows bleeding into (d) rectus sheath (arrow), (e) iliopsoas (arrow), and (f) pectineus muscles (arrow).

A diagnosis of dermatomyositis was made based on the presence of typical rash, mechanic's hands, muscle weakness, elevated muscle enzymes, abnormal EMG/MRI studies, and probable interstitial lung disease. The patient was started on solumedrol 80 mg IV BID and empiric levofloxacin for possible pneumonia. Her CPKs downtrended and her facial and body rashes began to resolve but she remained weak with worsening hypoxia and dysphonia. Given the aggressive nature of her disease, she was given IVIG infusion on consecutive days beginning approximately six days after admission while awaiting a planned muscle biopsy. She had a CT abdomen and pelvis, to search for possible malignancy during the second day of her IVIG infusion, which was read as negative for acute pathology (Figures 8(a)–8(c)). One day after finishing her second dose of IVIG, she became altered with a rapid drop of Hgb from 10.3 to 6.1 gm/dL. A repeat CT scan of the abdomen and pelvis revealed new areas of intramuscular hematomas that were not seen 24 hours ago on the prior CT (Figures 8(d)–8(f)). During her hospitalization, she had only been on prophylactic doses of subcutaneous dalteparin of 5000 units/day with a normal platelet count of 176 on the day of the bleeding event with an INR of 1.21 seconds on admission. Steroid therapy was increased to full 1-gram pulse doses and one dose of IV Cytoxan was given, but eventually the patient slowly decompensated and died of multiorgan failure a week later. A sample of her serum early in her hospital course was sent to a reference lab where notably she tested positive for anti-Ro/SSA-52 antibodies.

## 4. Discussion

Here we present two cases of idiopathic inflammatory myopathy (dermatomyositis) complicated by acute severe hemorrhagic myositis. The diagnosis of hemorrhagic myositis is rare and has been reported only several times in the literature but is a serious, often fatal, complication that has been associated with inflammatory myopathies (Table 1). Orrell et al. in a paper in 1998 discussed two cases in Rochester, NY, where patients with DM developed abdominal hematomas [7]. In the first case, a patient with biopsy proven DM for 11 years was started on IVIG monthly for active DM despite steroids and azathioprine. Five years later while on this regimen she developed acute left upper abdominal pain with Hgb drop from 12.9 to 9.1 g/dL and was found to have a large left rectus abdominis muscle hematoma. The patient did well with blood transfusion. The second patient was 11 years old with stable DM diagnosed at the age of 8 who presented with pain in the right hip for 3 days. She was taken to the OR for presumed appendicitis but was found to have retroperitoneal bleeding located from a hematoma posterior to the ascending colon and did well after surgery. A third case report by Fang et al. in Kaohsiung, Taiwan, 2008, described a 65-year-old female patient with polymyositis who developed retroperitoneal bleeding from the superior duodenal-pancreatic artery after a flare of her myopathy [8]. Although the bleeding stabilized with angiography, rebleeding occurred in the superior mesenteric artery that was again stabilized by angiography, but the patient expired from sepsis 10 days later. A fourth case presented by Yamagishi et al. in Niigata, Japan, in 2008 described a 64-year-old female with newly diagnosed DM with ILD who developed bleeding in her right psoas and iliacus muscles after 10 days of treatment with pulse steroids, oral cyclosporine, and IV cyclophosphamide [9]. Angiography localized the lesions to small aneurysms in the retroperitoneum that were embolized, with rebleeding occurring a week later that was again stopped by angiography, but the patient expired weeks later from TTP and multiorgan failure. Other case reports exist in the literature for hemorrhagic myositis, with a report from Higashi et al. in 2009 on a 77-year-old patient with DM who was on heparin for unstable angina [10].

Both of the cases presented in our study were consistent with dermatomyositis (and probably the antisynthetase syndrome) based on clinical features of rash, mechanic's hands, muscle weakness, ILD, elevated muscle enzymes, abnormal EMG/MRI (Case 2 only), and positive antibodies to anti-RO/SSA-52 from the same reference lab. The sites of bleeding in Case 1 (iliopsoas) and in Case 2 (rectus sheath and iliopsoas) were areas frequently reported in the other case reports. The finding of bleeding frequently in these areas but not in other muscle groups seems to be a possible unique finding in hemorrhagic myositis with inflammatory myopathy.

Both patients were given IVIG infusion for rapidly progressive disease. In Case 1, IVIG preceded the bleeding by several days and in Case 2 it followed directly the next day after IVIG infusion. In the past case reports, only one patient was receiving IVIG and had been doing so monthly for 5 years before the bleeding event. Reports of IVIG causing massive intravascular hemolysis are known but are rare, with the usual hemolysis reported as mild and self-limited [11]. Review of the literature did not show an association with IVIG bleeding into the muscle. More frequently reported IVIG complications include renal failure, anaphylaxis, aseptic meningitis, and thrombosis (up to 13% in one series of patients with autoimmune disorders) [12]. In that same review, it was notable that IVIG thrombotic events favored more arterial involvement (MI, CVA, and peripheral arteries) perhaps hinting at a possible role for enhanced arterial clotting or injury that could relate to hemorrhagic events in our cases.

Finally, both of our cases tested positive for anti-Ro/SSA-52 antibody, which is included among the myositis associated antibodies (MAA) [13]. Among autoantibodies in autoimmune disease, the Ro/SSA (made up of Ro60 and Ro52) antibody is the most common one to extractable nuclear antigens. Antibodies to Ro-52 (a tripartite motif protein/TRIM) can be found independent of antibodies to Ro-60 (a small cytoplasmic ribonucleoprotein/scRNP) in certain autoimmune disorders. Expression of isolated anti-Ro52 alone is mainly seen in certain connective tissue diseases (including myositis), but with unclear clinical significance with an overall prevalence of 0.5% [14]. The anti-Ro52 antibody occurs in about 30% of patients with inflammatory myopathy and up to 72% of those that are also positive for anti-Jo-1, a myositis specific antibody (MSA) associated with ILD. However, since both our cases were negative for selected MSAs including anti-Jo-1, it is unclear whether the positive testing for anti-Ro52 may be associated with inflammatory myopathy and ILD or possibly overlapping with additional, nondetected antibodies more specific for inflammatory myopathy associated with this serious complication.

## 5. Conclusion

In summary, we present two cases of inflammatory myopathy (dermatomyositis with ILD) complicated by severe hemorrhagic myositis. The pattern of bleeding in our cases, as in the previous literature, seems to favor the retroperitoneum or rectus abdominis muscles. The possibility of hemorrhagic myositis should always be entertained as a possible complication of inflammatory myopathy, especially in the setting of an acute drop in the hemoglobin. It remains to be seen if the anti-Ro52 antibody may play an accessory role in predicting which patients may be at risk for this potentially very serious complication.

## Figures and Tables

**Table 1:** Clinical features of selected case reports of hemorrhagic myositis in inflammatory myopathies.

Author	Age (yrs) (at event)	Gender	Clinical history and prebleeding therapies	Antibody profile	Site of bleeding(s)	Treatment	Outcome (as reported)
Orrell et al. [7]	50	Female	DM age 34 Corticosteroid Azathioprine IVIG	None specified	Left rectus abdominis	Blood transfusions Prednisone Azathioprine	Survived, no recurrent bleeding

Orrell et al. [7]	11	Female	DM age 8 Prednisone	ANA 1 : 320 speckled	Retroperitoneum, posterior to right ascending colon	Supportive	Survived without recurrence

Fang et al. [8]	65	Female	PM age 65 Hydrocortisone Prednisone	ANA 1 : 320 speckled	Retroperitoneum, superior duodenal pancreatic artery, SMA	Angiography with embolization	Expired from sepsis without rebleeding

Yamagishi et al. [9]	64	Female	DM/ILD age 64 Solumedrol Prednisone Cyclosporine Dalteparin	ANA positive Elevated serum KL-6	Retroperitoneum, right psoas, right iliacus, left rectus sheath	Angiography with embolization with rebleeding and repeat embolization	Expired from TTP and multiorgan failure

Higashi et al. [10]	77	Female	DM age 77 Solumedrol Prednisone Cyclosporine Heparin	Elevated serum KL-6	Retroperitoneum, left iliopsoas, left iliacus, right sternomastoid	Supportive	Expired from DIC

Van Gelder et al.	50	Male	DM/ILD age 50 Solumedrol IVIG	Anti-Ro52	Retroperitoneum, right iliacus, right psoas	Transfusions Solumedrol	Expired from multiorgan failure

Van Gelder et al.	63	Female	DM/ILD age 63 Solumedrol IVIG	Anti-SSA Anti-Ro52	Retroperitoneum, right pectineus, right iliopsoas	Transfusions Solumedrol Cytoxan	Expired from multiorgan failure
